# Gestational and Lactational Exposure to BPS Triggers Microglial Ferroptosis via the SLC7A11/GPX4 Antioxidant Axis and Induces Memory Impairment in Offspring Mice

**DOI:** 10.3390/ijms262411953

**Published:** 2025-12-11

**Authors:** Nuo Xu, Xinxin Guo, Yan Su, Mengfen Pan, Kaixing Lin, Zhensong Ma, Haozhe Zhou, Huaicai Zeng

**Affiliations:** 1 Guangxi Key Laboratory of Environmental Exposomics and Entire Lifecycle Health, School of Public Health, Guilin Medical University, Guilin 541199, China; 2 Guangxi Health Commission Key Laboratory of Entire Lifecycle Health and Care, School of Public Health, Guilin Medical University, Guilin 541199, China

**Keywords:** bisphenol S, microglia, ferroptosis, SLC7A11/GPX4, neurotoxicity

## Abstract

This study aimed to examine the role of maternal BPS gestational and lactational exposure to BPS in neurotoxicity in offspring *mice* and to uncover the regulatory mechanisms driven by microglial ferroptosis. In this study, C57BL/6J *mice* were treated with BPS during pregnancy and lactation. The results revealed that BPS induced memory impairment and anxiety in offspring *mice*, accompanied by abnormal expression levels of brain neurotrophic factor and synaptic plasticity factor (PSD95, SYP). Additionally, exposure to BPS activated microglia by upregulating the expression of IBA1 and concurrently promoting the release of inflammatory factors in the hippocampus and cortex. BPS exposure also contributed to iron overload, aberrant mitochondrial morphology, oxidative stress, and abnormal expression of ferroptosis-associated genes (GPX4, SCL7A11, TFR1, ACSL4) in the brains of offspring *mice*. Importantly, immunofluorescence analysis demonstrated concomitant microglial activation and ferroptosis in the brain tissue of offspring *mice* following BPS exposure. Moreover, experiments in BV2 microglial cells showed that the ferroptosis inhibitor Fer-1 reversed BPS-induced microglial ferroptosis and the release of inflammatory cytokines. These findings collectively elucidate the regulatory role mechanism of microglial ferroptosis in BPS-induced neurotoxicity in offspring *mice*, and we propose potential therapeutic targets for attenuating BPS-mediated neurotoxic effects.

## 1. Introduction

Bisphenol S (BPS), as a primary substitute for bisphenol A (BPA), has been extensively used in food-contact materials, medical equipment, and infant products [1,2]. At present, it is increasingly detected across global environmental media, food products, and human biological samples [3,4]. Earlier studies have established that infancy represents a crucial window for neural development. Environmental pollutants such as BPS can infiltrate the placental barrier during gestation and lactation, adversely affecting offspring neurodevelopment [5]. Mounting evidence from epidemiological research suggests that prenatal exposure to BPS disrupts neurodevelopmental processes in children [6]. Additionally, animal experiments have revealed that developmental BPS exposure leads to sexually dimorphic behavioral changes, characterized by heightened anxiety in male offspring and decreased exploration in female offspring [7]. Infancy and childhood represent critical periods for neurodevelopment, and the effects of BPS exposure during these periods and its underlying mechanisms require systematic investigation.

Microglia, the earliest maturing glial cells in the central nervous system, function as resident macrophages within the brain. They participate in numerous key processes, including synaptic pruning, neural circuit refinement, and the regulation of brain development and neuronal network maintenance [8]. Environmental toxins can induce microglial activation and neuroinflammation, thereby disrupting neurodevelopment and contributing to the pathogenesis of neurodegenerative diseases [9]. Meanwhile, a prior investigation has indicated that inflammatory activation of microglia can stimulate naturally neurotrophic astrocytes to transition into a neurotoxic state [10]. Notably, hyperreactive microglia of the M1 pro-inflammatory subtype lead to pathological tau phosphorylation, sustained neuroinflammation, and subsequent neuronal degeneration [11]. Under the pathological conditions of neurodegenerative diseases, iron overload triggers the polarization of microglia into the pro-inflammatory (M1) phenotype through ROS, thereby facilitating the secretion of TNF-α and promoting neuroinflammation [12]. TNF-α upregulates TFR1 and DMT1 and downregulates FPN, thereby simultaneously increasing ferrous influx and limiting intracellular iron exocytosis [13]. Therefore, the interaction between neuroinflammation and iron forms an amplified ROS production circuit, eventually culminating in neuronal iron death. Overall, both inflammatory cell death and ferroptosis serve as critical mechanisms underlying neurodevelopmental impairments and neuronal death [14]. Within the brain, microglia exhibit the highest capacity for iron accumulation among all neural cells. Microglia-derived pro-inflammatory mediators further promote iron overload, which in turn drives iron accumulation, ultimately leading to ferroptosis and consequent cognitive deficits [15].

Recently, a large number of studies have demonstrated that ferroptosis, a newly discovered type of programmed cell death, plays a central role in pollutant-induced toxicity [16]. An in vitro study also indicated that ferroptosis plays a significant role in BPA-induced neurotoxicity and represents a candidate target for the treatment of various neurological diseases [17]. Existing studies have shown that bisphenol A induces placental ferroptosis and fetal growth restriction through the YAP/Taz-ferritin phagocytic axis [18]. Previous studies have concluded that the release of lipid metabolites during ferroptosis triggers inflammation, which in turn promotes ferroptosis by increasing iron deposition [19]. Of note, exposure to BPS led to an increase in the level of oxidative stress in the fetal brain [20]. However, the role of ferroptosis in BPS-induced neurodevelopmental toxicity remains elusive. Therefore, this study aimed to investigate the effects of gestational and lactational BPS exposure to BPS on fetal brain development and behavioral outcomes and to determine the contribution of microglia-mediated inflammatory ferroptosis to BPS-induced neurodevelopmental toxicity in offspring *mice*.

## 2. Results

### 2.1. Perinatal BPS Exposure Induces Cognitive Deficits and an Anxiety-like Phenotype in Offspring Mice

**Figure 1:**
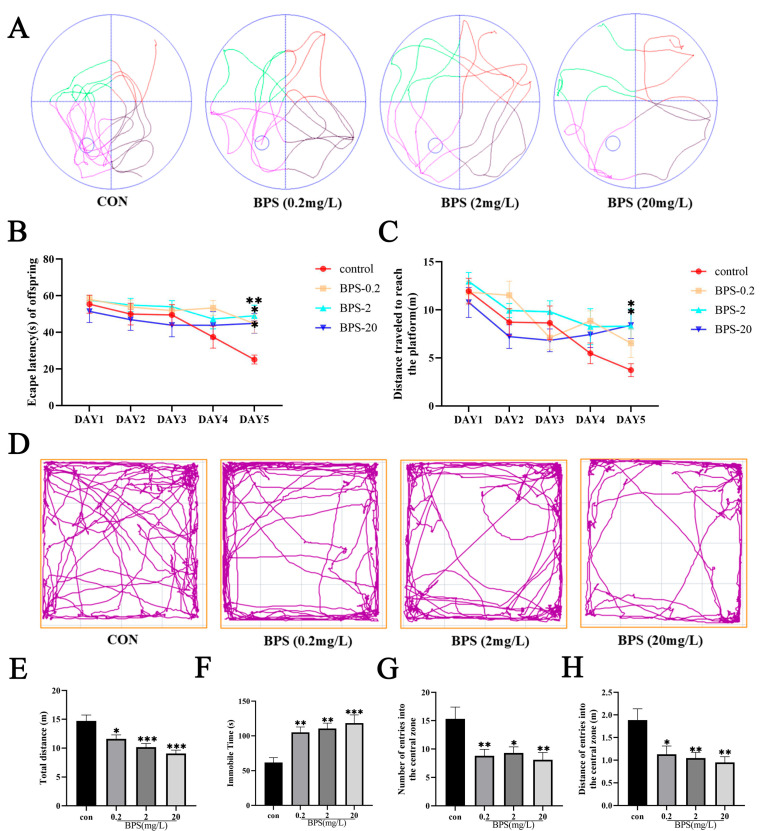
Perinatal BPS exposure induces cognitive deficits and an anxious phenotype in offspring *mice*. (**A**) Representative swimming paths of offspring in space detection experiments. (**B**) Escape latency in spatial learning experiments of offspring *mice*. (**C**) Distance traveled by offspring to reach the platform for the first time. (**D**) Representative track records of offspring during OFT. (**E**) Total distance traveled by offspring during OFT. (**F**) Immobile time of offspring during OFT. (**G**) Number of entries into the central zone. (**H**) Distance of entries into the central zone. Data are expressed as mean ± standard error (*n* = 8–10). * *p* < 0.05, ** *p* < 0.01, *** *p* < 0.001.

The MWM test employed evaluated learning and memory performance in offspring *mice* following perinatal BPS exposure. During the first 5 days, spatial learning ability was tested. During this period, the escape latency and swimming distance of offspring *mice* upon their first arrival at the platform progressively decreased (Figure 1A–C). In addition, compared with the control group, there were significant differences on day 5 (Figure 1B, *p* < 0.05). Likewise, the swimming distance of offspring *mice* in the BPS-treated (2 and 20 mg/L) groups when they first reached the platform on the fifth day was significantly lower than the control group (Figure 1C, *p* < 0.05). These results conjointly suggest that exposure to BPS during pregnancy and lactation may impair the spatial learning and memory ability of the offspring.

In the OFT, *mice* with lower anxiety levels tend to spend more time in the center arena of the arena compared to the border to explore the open space [21]. Compared with controls, BPS-exposed *mice* exhibited a shorter swimming distance and a longer immobility duration (Figure 1D–F, *p* < 0.05), reflecting that exposure to BPS during pregnancy and lactation significantly limited locomotor activity in offspring *mice*. Additionally, both the number of entries into and the distance traveled in the central zone were significantly decreased in all BPS-treated groups (Figure 1G,H, *p* < 0.05). Taken together, these results suggested that offspring *mice* developed anxiety-like behavioral phenotypes following BPS exposure during pregnancy and lactation periods.

### 2.2. Perinatal BPS Exposure Impairs Hippocampal Synaptic Integrity in Offspring Mice

**Figure 2:**
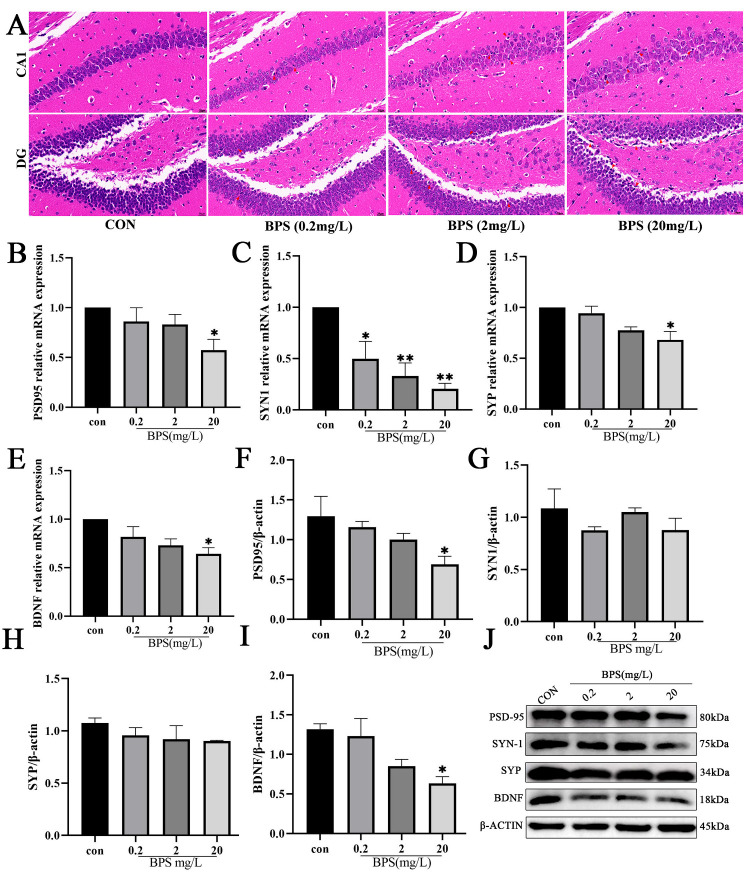
Perinatal BPS exposure impairs hippocampal synaptic integrity in offspring *mice*. (**A**) HE staining images of the hippocampus of offspring mice (CA1: magnification: 63×, bar = 20 μm; DG: magnification: 63×, bar = 20 μm). The red arrows represent neurons with nuclear shrinkage. (**B**–**E**) mRNA level of *BDNF*, *PSD95, SYP*, and *SYN1*. (**F**) Quantitative results of blotting of PSD95. (**G**) Quantitative results of blotting of SYN. (**H**) Quantitative results of blotting of SYP. (**I**) Quantitative results of blotting of BDNF. (**J**) WB electrophoresis bands. Data are expressed as mean ± standard error (*n* = 3). * *p* < 0.05, ** *p* < 0.01.

To further investigate the impact of BPS exposure during pregnancy and lactation on brain tissue injury in offspring *mice*, HE staining was performed to observe pathological alterations. As illustrated in Figure 2A, hippocampal neurons in the CA1 and DG regions of the control group were regularly and tightly arranged during under light microscopy. In contrast, BPS exposure significantly reduced the number of CA1 pyramidal cell layers, induced morphological alterations characterized by indistinct nuclear boundaries, and led to dispersed and irregularly arranged granule neurons in the DG region. Collectively, these results indicate that BPS exerts adverse effects on the hippocampal morphology in offspring.

Then, RT-qPCR and WB analyses were conducted to further validate BPS -induced hippocampal injury at the transcriptomic and protein levels. As anticipated, the results of RT-qPCR showed that the mRNA expression levels of *BDNF*, *PSD95, SYP*, and *SYN1* were lower in all BPS-treated groups compared with the control group (Figure 2B–E), with the most pronounced reduction observed in the 20 mg/L BPS group (*p* < 0.05). Likewise, the results of WB analysis revealed that compared with the control group, the protein levels of PSD95 and BDNF were significantly lower in the 20 mg/L BPS group compared with the control group (Figure 2F–J, *p* < 0.05). The aforementioned results conjointly indicate that gestational and lactational BPS exposure leads to synaptic impairment in offspring mice, consequently resulting in behavioral deficits.

### 2.3. BPS Exposure During Pregnancy and Lactation Leads to Iron Accumulation and Oxidative Stress in the Brain of Offspring Mice

**Figure 3:**
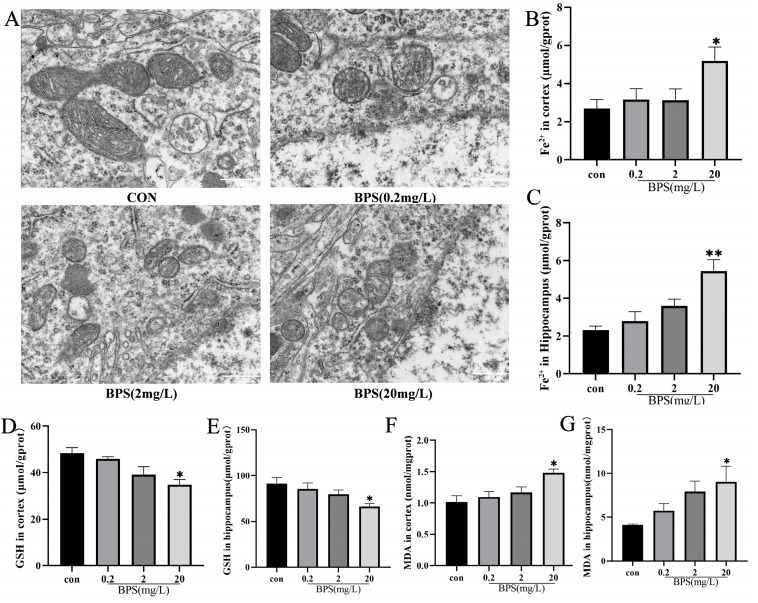
BPS exposure during pregnancy and lactation leads to iron accumulation and oxidative stress in the brains of offspring *mice*. (**A**) Ultrastructural morphology of hippocampal mitochondria in offspring *mice*. Neuronal images were acquired using a Hitachi TEM system, ×20 k, bar = 500 nm. (**B**,**C**) The effect of BPS on iron content in the cortex and hippocampus. (**D**,**E**) Effects of BPS on GSH levels in the cortex and hippocampus of offspring *mice*. (**F**,**G**) Effect of BPS on MDA levels in the cortex and hippocampus of offspring *mice*. Data are expressed as mean ± standard error (*n* = 3). * *p* < 0.05, ** *p* < 0.01.

To further explore the mechanism by which BPS causes brain injury in offspring, phenotypic features of ferroptosis were assessed in the cerebral tissues of offspring mice. TEM revealed significant ultrastructural abnormalities in the hippocampus of BPS-exposed offspring, characterized by mitochondrial atrophy, aberrant chromatin distribution, and disruption or loss mitochondrial cristae (Figure 3A). Furthermore, iron assays demonstrated a significant reduction in iron content in the hippocampal and cortical tissues of BPS-exposed offspring *mice* (Figure 3B,C, *p* < 0.05). More importantly, exposure to 20 mg/L BPS induced a significant reduction in GSH levels, accompanied by a concurrent increase in MDA levels in the offspring hippocampus and cortex of offspring *mice* (Figure 3D–G, *p* < 0.05). Taken together, these results indicate that gestational and lactational BPS exposure induces iron accumulation and oxidative stress in the brains of offspring *mice*.

### 2.4. Pregnancy and Lactation BPS Exposure Induces Ferroptosis in the Brains of Offspring Mice

**Figure 4:**
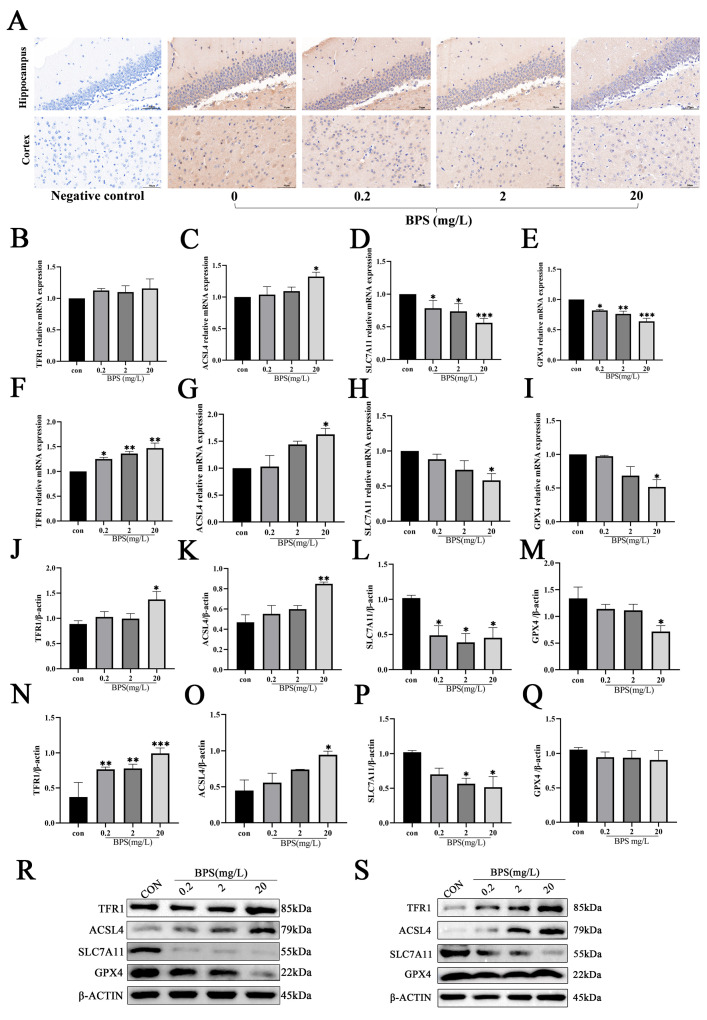
Pregnancy and lactation BPS exposure induces brain ferroptosis in offspring *mice*. (**A**) The expression of GPX4 in the hippocampus and cortex in offspring *mice*, 40×, bar = 50 μm. (**B**–**E**) The mRNA levels of *TFR1*, *ACSL4*, *SLC7A11*, and *GPX4* in the hippocampus. (**F**–**I**) The mRNA levels of *TFR1*, *ACSL4*, *SLC7A11*, and *GPX4* in the cortex. (**J**–**M**) Quantitative results of Western blotting of TFR1, ACSL4, SLC7A11, and GPX4 in the hippocampus. (**N**–**Q**) Quantitative results of Western blotting of TFR1, ACSL4, SLC7A11, and GPX4 in the cortex. (**R**) WB electrophoresis bands of the hippocampus. (**S**) WB electrophoresis bands of the cortex. Data are expressed as mean ± standard error, *n* = 3. * *p* < 0.05, ** *p* < 0.01, *** *p* < 0.001.

To further investigate the degree to which gestational and lactational BPS exposure induces ferroptosis in the brain tissue of offspring *mice*, we examined relevant ferroptosis-related markers. As displayed in Figure 4B–I, RT-qPCR showed that the mRNA levels of *TFR1* and *ACSL4* were higher, whereas those of *GPX4* and *SLC7A11* were lower, in the 20 mg/L BPS groups compared to the control group (Figure 4B–I, *p* < 0.05). At the same time, the results of WB analysis demonstrated that the protein levels of TFR1 and ACSL4 were significantly higher in the hippocampus and cortex of the 20 mg/L BPS group compared with the control group, whereas the protein levels of SLC7A11 and GPX4 were significantly lower in the hippocampus and cortex of the 20 mg/L BPS group (Figure 4J–S, *p* < 0.05). Immunohistochemical analysis delineated that compared with the untreated offspring *mice*, the expression level of GPX4 in the hippocampus and cortex of offspring *mice* treated with BPS (Figure 4A) was significantly decreased in a dose-dependent manner. Collectively, these findings demonstrate that perinatal BPS exposure induces ferroptosis by modulating SLC7A11 and GPX4 expression in the offspring hippocampus and cortex at both transcriptional and translational levels.

### 2.5. Pregnancy and Lactation BPS Exposure Induces Ferroptosis and Activates Microglia in Offspring Mice

**Figure 5:**
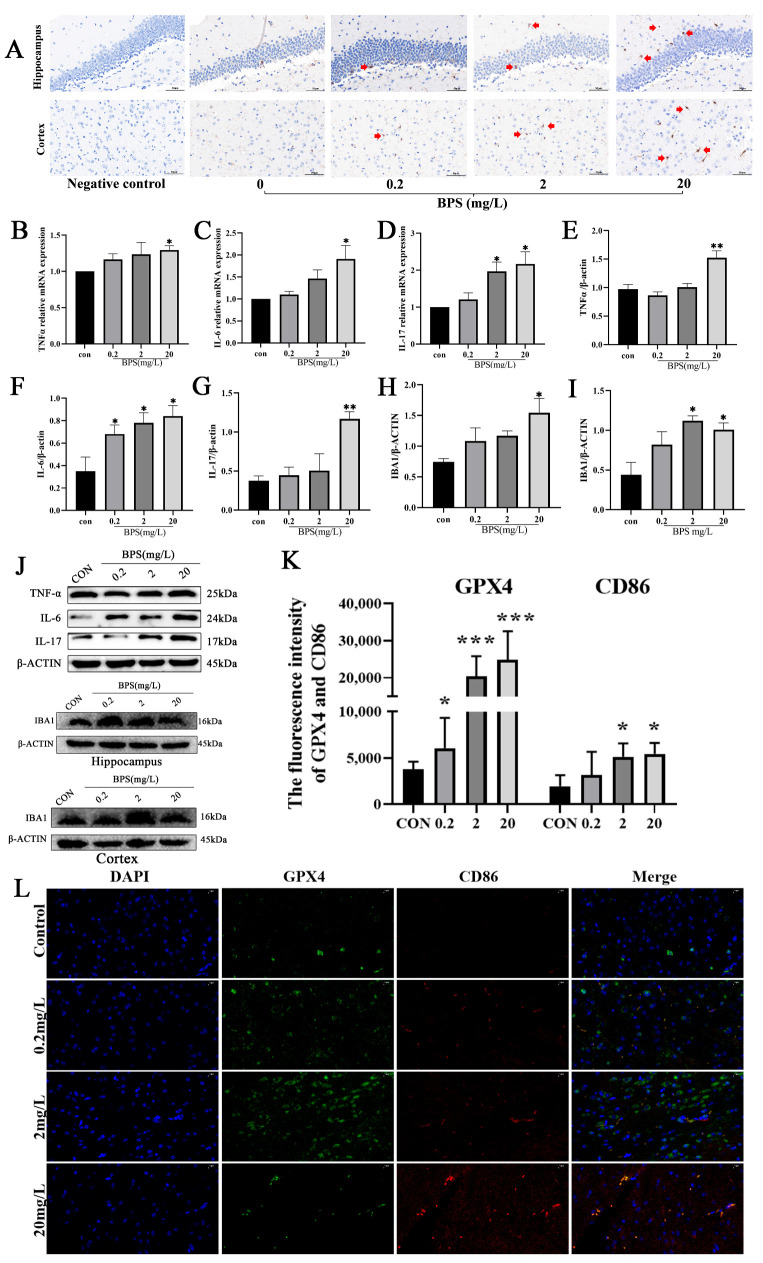
Pregnancy and lactation BPS exposure induced ferroptosis and activated microglia in offspring *mice*. (**A**) The expression of IBA1 (red arrow) in the cortex and hippocampus. Scale = 50 μm. (**B**–**D**) The mRNA levels of *TNF-α*, *IL-6*, and *IL-17* in the cortex. (**E**–**G**) Quantitative results of Western blotting of TNF-α, IL-6, and IL-17 in the cortex. (**H**,**I**) Quantitative results of Western blotting of IBA1 in the hippocampus. (**J**) WB electrophoresis bands of the cortex and hippocampus. (**K**) The fluorescence intensity of GPX4 and CD86. (**L**) The expression of GPX4 (green) and CD86 (red, markers of M1-type microglia) in the cortex, 40×, bar = 20 μm. Data are expressed as mean ± standard error, *n* = 3. * *p* < 0.05, ** *p* < 0.01, *** *p* < 0.001.

Previous studies have documented that iron overload drives microglial toward polarization toward the pro-inflammatory (M1) phenotype through ROS, thereby promoting neuroinflammatory responses [22]. Herein, an increased number of microglia were detected in the hippocampus and cortex of *mice* in the BPS-exposed group compared with the control group (Figure 5A). Moreover, the expression level of IBA1 was detected in the cerebral cortex and hippocampus via WB analysis to assess the activation state of microglia. Interestingly, the results signaled that BPS exposure during pregnancy and lactation upregulated IBA1 expression in the cerebral cortex and hippocampus of offspring *mice* (Figure 5H–J, *p* < 0.05). At the same time, the levels of several markers of microglial M1 polarization were quantified, and the results showed that the *TNF-α*, *IL-6*, and *IL-17* mRNA levels were significantly higher in the 20 mg/L BPS group compared with the control group (Figure 5B–D) (*p* < 0.05). Similarly, WB analysis also showed that TNF-α, IL-6, and IL-17 protein levels were significantly higher in the 20 mg/L BPS group compared with the control group (Figure 5E–G, *p* < 0.05). Meanwhile, we also found that the expression of CD86 was upregulated, whilst that of GPX4 was downregulated, in the brain tissue of offspring *mice* following gestational and lactational BPS exposure, (Figure 5K,L). Noteworthily, ferroptosis manifestation appeared to be temporally associated with microglial activation. These results suggest that BPS exposure during pregnancy and lactation may promote ferroptosis in the brains of offspring *mice*, thereby inducing microglial activation and the release of inflammatory mediators, ultimately leading to neurotoxicity.

### 2.6. Fer-1 Inhibited the Release of Inflammatory Factors in BV2 Cells

**Figure 6:**
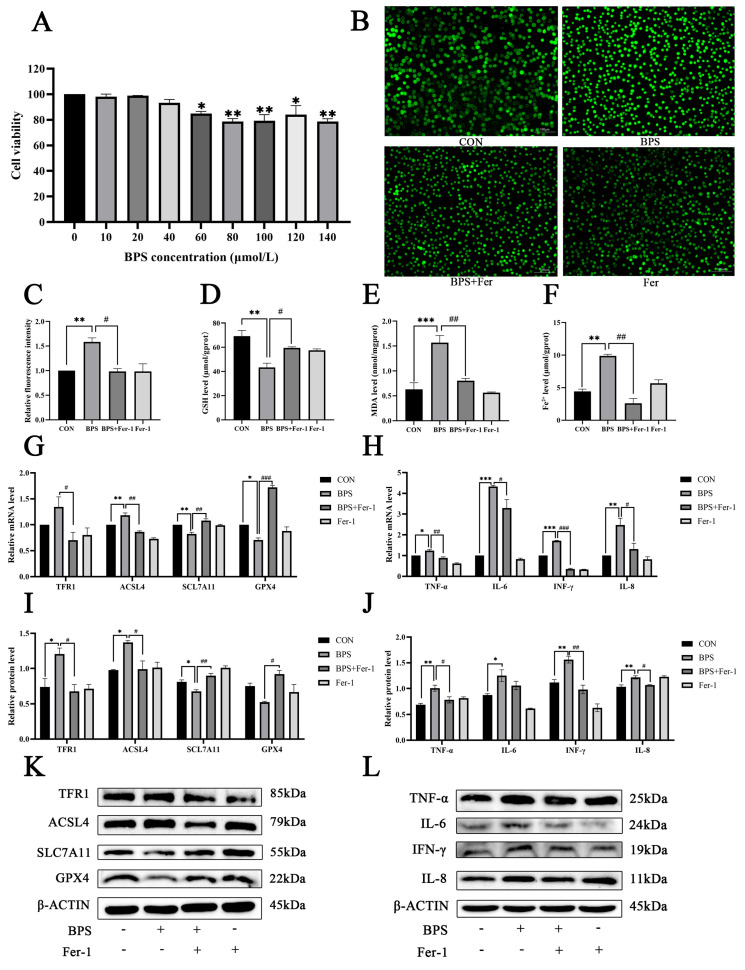
Fer-1 inhibited the release of inflammatory factors in BV2 cells. (**A**) The effect of different concentrations of BPS on the viability of BV2 cells. (**B**) ROS visualized by fluorescence. (**C**) Quantitative ROS using Image J. (**D**–**F**) The contents of GSH, MDA, and iron in BV2 cells. (**G**) The mRNA levels of *TFR1*, *ACSL4*, *SCL7A11*, and *GPX4* in BV2 cells. (**H**) The mRNA levels of TNF-α, IL-6, INF-γ, and IL-8 in BV2 cells. (**I**) Quantitative results of WB of TFR1, ACSL4, SCL7A11, and GPX4 in BV2. (**J**) Quantitative results of WB of TNF-α, IL-6, INF-γ, and IL-8 in BV2. (**K**) WB electrophoresis band for ferroptosis biomarkers. (**L**) WB electrophoresis band for inflammatory factor. The data is expressed as the mean ± standard error, *n* = 3. * *p* < 0.05, ** *p* < 0.01, *** *p* < 0.001, ^#^ *p* < 0.05, ^##^ *p* < 0.01, ^###^ *p* < 0.001.

To explore this hypothesis, an in vitro BV2 microglial cell model was established. Under these experimental conditions, exposure to BPS at concentrations exceeding 60 μmol/L for 24 h reduced the cell viability of BV2 cells (Figure 6A, *p* < 0.05). Pretreatment with Fer-1 attenuated ROS and MDA generation while concurrently increasing the GSH levels (Figure 6B–E, *p* < 0.05). Compared with the control group, the level of Fe^2+^ in the cytoplasm of BV2 cells was significantly higher in the BPS-treated group, while Fer-1 restored the content of Fe^2+^ toward baseline levels (Figure 6F, *p* < 0.05). Four widely reported ferroptosis biomarkers were assessed in the BV2 cells, and the results showed that BPS upregulated the expressions of TFR1 and ACSL4 and significantly downregulated those of GPX4 and SLC7A1 (Figure 6G, *p* < 0.05). However, Fer-1 reversed these alterations and restored the expression of all key proteins (Figure 6I–L, *p* < 0.05). These findings demonstrate that BPS exposure induces ferroptosis in microglia. Pretreatment with Fer-1 almost completely normalized the BPS-induced increase in the levels of pro-inflammatory cytokines TNF-α, IL-6, IFN-γ, and IL-8 in BV2 cells, indicating that ferroptosis inhibition redirects microglia away from the M1-like phenotype (Figure 6G–L, *p* < 0.05).

## 3. Discussion

The present study employed an in vivo mouse model of BPS exposure to investigate its impact on offspring neurodevelopment following gestational and lactational exposure and established an in vitro BV2 cell model to elucidate the roles and mechanisms underlying BPS-mediated neurofunctional impairment. The results revealed that exposure to BPS during the period from GD0 to PND21 resulted in learning and memory deficits, as well as anxiety-like behaviors, in offspring *mice*. Furthermore, BPS treatment triggered ferroptosis and oxidative stress, as well as microglial activation, in the brains of the offspring.

Numerous studies have pointed out that exposure to BPS can lead to neurotoxicity and neurobehavioral disturbances, resulting in memory impairment, and synaptic dysfunction [23]. The present study validated the theory that early exposure to BPS reduced learning and memory and motor abilities, as well as eliciting anxiety-like behaviors, in adult offspring. Furthermore, BPS exposure elicited nuclear shrinkage of the hippocampal neurons and downregulated the expression of genes (BDNF, PSD-95, and SYP) related to synaptic function. As a pivotal neurotrophic factor, BDNF plays an essential role in embryonic neural development, cell survival, and synaptic pruning [24]. A previous study indicated that prenatal alcohol exposure disrupts CREB/BDNF/TrkB signaling through oxidative stress, thereby impairing cognitive function in offspring [25]. Our earlier studies have consistently established BDNF as a key regulator of neurological function [26,27]. PSD-95 is the most abundant protein in postsynaptic density and plays crucial roles in neurotransmission, synaptic plasticity, and dendritic morphology during neurodevelopment [28]. SYN serves as a presynaptic marker whose expression level accurately reflects synaptic density and further indicates the state of synaptogenesis [29]. Consistent with the present findings, reduced expression of PSD-95 and SYN impairs synaptic function and plays a decisive role in fluoride-induced neurodevelopmental toxicity [30].

Microglia are embryonically derived tissue-resident macrophages within the parenchyma of the central nervous system and have long been recognized as neuronal circuit sculptors implicated in neurodevelopment. Beyond shaping neuronal circuits during both developmental and adult stages, they also perform immune surveillance, supply trophic support for neurogenesis, preserve the integrity of the blood–brain barrier, and directly modulate neuronal activity [31]. Similarly, neuroinflammation has been shown to contribute to the neurodegenerative effects of BPS [32]. A study inferred that early-life exposure to BPA impaired neurobehavioral outcomes in adult offspring, accompanied by excessive activation of hippocampal microglia [33]. Microglial activation and the subsequent release of inflammatory factors (TNF-α and IL-6) are key contributors to anxiety and depression-like phenotypes [11,34]. A review evinced that IL-6 can regulate several important neuronal and synaptic processes, including synaptic transmission and synaptic plasticity, which constitute a central cellular mechanism underlying memory and learning [35]. Another study indicated that BV2 microglial cells, which perform key immune functions including surveillance, phagocytosis, cytokine release, and response to central nervous system injury, are implicated in manganese-induced neurotoxicity [36]. This study corroborated that BPS exposure induced the activation of microglia (IBA1) and the release of TNF-α, IL-6, and IL-17 in the brains of offspring *mice*, consistent with the findings of previous studies.

In addition, ferroptosis plays a vital role not only in neurodevelopment but also in the neuroinflammatory processes that disrupt cognitive function in offspring *mice* [37]. In vivo studies have described that TPhP exposure induces oxidative stress and ferroptosis, thereby causing neurodevelopmental toxicity in zebrafish [38]. Herein, BPS exposure resulted in atrophic hippocampal mitochondria, ruptured mitochondrial cristae, and complete crista loss. Exposure to BPS also promotes Fe^2+^ accumulation, decreases GSH levels, and increases MDA levels, which are hallmarks of ferroptosis [39]. A recent study indicated that iron overload triggers microglial polarization toward the pro-inflammatory (M1) phenotype via ROS, thereby promoting the secretion of TNF-α and IL-1β, and promotes neuroinflammation [12]. Moreover, microglia-mediated neuroinflammation and iron driven by gp91phoxhave been implicated in rotenone-induced learning and memory disorders in *mice* [34]. Using an in vitro BV2 cell model co-treated with the ferroptosis inhibitor Fer-1 and BPS, this study demonstrated that Fer-1 alleviated ferroptosis in BV2 cells by minimizing BPS-induced elevations in ROS and MDA levels, as well as Fe^2+^ overload, while attenuating the BPS-mediated decrease in GSH levels and restoring the expression of ferroptosis-related genes. Furthermore, the inhibition of ferroptosis subsequently inhibited the release of inflammatory factors (TNF-α, IL-6, and IL-17) from BV2 cells, suggesting that the inflammatory response elicited by BPS is closely related to ferroptosis.

This study proposes that gestational or lactational exposure to BPS impaired neurodevelopment by altering the expression of synaptic-related proteins, while BPS also induces ferroptosis and microglial activation in offspring *mice*. Through a sequential strategy of an in vitro experiment followed by targeted ferroptosis inhibition using Fer-1, the current study established compelling evidence supporting microglial ferroptosis as a key mechanism underlying BPS-induced neurodevelopmental impairment. Nevertheless, it is worth acknowledging that there was no additional intervention, and the interaction and relationship between inflammation and ferroptosis in BPS-induced neurodevelopmental toxicity was not systematically explored. While the findings of this study indicate a role for ferroptosis, the absence of targeted in vivo interventions precludes definitive conclusions regarding its predominance among potential contributing factors in the in vivo experiment, and the underlying mechanisms also warrant further investigation.

## 4. Materials and Methods

### 4.1. Animals and Animal Experiments

Female and male wild-type C57BL/6 *mice* (4 weeks old, 16–18 g) were sourced from Hunan SJA Laboratory Animal Co., Ltd. (SCXK (Xiang) 2019-0004, Changsha, China) and kept under standard laboratory conditions (20–25 °C, 30–50% humidity, 12-h light/dark cycle) with ad libitum access to food and water. After 1 week of acclimatization, male and female *mice* were cage-mated at a ratio of 2:1 at 20:00 daily, and vaginal plug formation was checked at 8:00 the next day. The detection of vaginal plugs was denoted as GD 0. Next, pregnant *mice* were randomly divided into 4 groups, with 10 *mice*. From GD 0 day to PND 21, pregnant *mice* were treated with different concentrations of BPS (0, 0.2, 2, 20 mg/L) through drinking water daily. Offspring *mice* (PND 22–56) were provided with distilled water and food ad libitum. After 56 days [40], offspring *mice* were harvested and stored at −80 °C. In addition, 1 cm × 1 cm × 1 cm tissue sections were fixed in 4% paraformaldehyde for subsequent experiments. All animal experiments were conducted in strict accordance with ARRIVE guidelines and the ethical guidelines outlined in the European Union Directive 2010/63/EU on the protection of animals used for scientific purposes. All experiments were approved by the Institutional Animal Care and Use Committee of Guilin Medical University (NO: GLMC-IACUC-202510101).

### 4.2. Hematoxylin and Eosin (H&E) Staining

The fresh brain tissues were fixed with 4% paraformaldehyde, dehydrated, and embedded in paraffin. Then, 4 μm thick brain sections were prepared and hydrated in xylene (2 times, each for 20 min) and rehydrated through a series of graded ethanol solutions (95%, 90%, 80%, 70%, and 50%). After staining with hematoxylin and eosin, the sections were dehydrated using anhydrous ethanol (three times, each for 5 min), and finally, the slides were rapidly air-dried and mounted with neutral gum. Lastly, the sections were observed and imaged using an inverted microscope (Nikon, Tokyo, Japan).

### 4.3. Open Field Test (OFT)

The open-field test was performed to assess anxiety-like behavior and locomotor. activity. Briefly, a 45 cm × 45 cm arena was used to assess *mice* (PND 56) as they wandered through space. *Mice* were placed in the same corner of the apparatus and allowed to explore freely for five minutes. The distance traveled, time and distance spent in the center, and distance in the center zone were recorded using ANY-maze 7.20 software [41]. *Mice* exhibiting anxiety-like behavior typically spend more time in the corners and edges than in the center.

### 4.4. Morris Water Maze Experiment (MWM)

The Morris water maze experiment forces experimental animals to swim and find platforms hidden in the water. It is mainly used to test the animals’ learning and memory abilities in terms of spatial position sense and direction sense. On PND 56, spatial learning and memory were assessed in a Morris water maze (150 cm diameter × 50 cm height) filled with opaque water maintained at 22 °C. The training stage lasts for 5 days, with 4 training sessions conducted each day. Each time, *mice* facing the pool walls are placed in the water from different quadrants. The escape delay period and the swimming path are recorded. The testing stages were as follows: the space exploration test was conducted on the sixth day of the experiment. The escape platform was removed, and rats were allowed to enter the water from the quadrant opposite the platform. The time that *mice* stay in the target quadrant and their swimming paths are recorded to evaluate their spatial memory ability.

### 4.5. Transmission Electron Microscopy (TEM)

Fresh brain tissues were sliced into 0.3 cm × 0.3 cm × 0.3 cm cubes and fixed in 2.5% glutaraldehyde solution (Beyotime, Shanghai, China) at 4 °C overnight. After washing with phosphate buffer 3 times, the tissues were fixed with 1% osmium tetroxide for 2 h. Thereafter, tissues were dehydrated through a graded ethanol series, following which ultrathin sections (<80 nm) were prepared using a microtome (Leica, Wetzlar, Germany). These sections were subsequently mounted on copper grids, stained with uranyl acetate and lead citrate, and then observed and photographed using a transmission electron microscope (TEM, Hitachi, HT7800, Tokyo, Japan).

### 4.6. Immunohistochemistry (IHC) Analysis

Paraffin-embedded brain sections were initially deparaffinized, followed by antigen retrieval using citric acid buffer (PH6.0). Endogenous peroxidase activity was blocked with 3% H_2_O_2_, followed by incubation with 5% BSA to reduce nonspecific binding. Afterward, the sections were incubated with primary antibodies (IBA-1, HUABIO, Hangzhou, China, ET1705-78, 1:1000; GPX4, Abcam, Cambridge, UK, AB125066, 1:1000) at 4 °C overnight. After washing three times with PBS, the sections were incubated with the secondary antibody (HRP-labeled goat anti-rabbit and mouse universal secondary antibody DAKO 1:1) at room temperature in the dark for one hour. Then, the cell nuclei were stained, counterstained, and re-stained with DAB, followed by dehydration and mounting. Images were captured under a light microscope (Pannoramic SCAN, 3D HISTECH, Budapest, Hungary) and analyzed with ImageJ software.

### 4.7. Cellular Culture and Viability Assay

BV2 cells were cultured in DMEM medium including fetal bovine serum and double antibodies in a volume ratio of 89:10:1. After daily passage, the cells were placed in an incubator at 37 °C and 5% CO_2_ for culture. During the administration process, BV2 cells were exposed to BPS staining solution prepared by dissolving BPS in DMEM at a dose of 40 µmol/L, and the inhibitor group was exposed to Fer-1 staining solution prepared by dissolving Fer-1 in DMEM at a dose of 0.5 μmol/L [34,42], while the control group was given DMEM medium and exposed for 24 h. In the experimental group with the inhibitor applied, the cells must be pretreated 2 h before exposure.

BV-2 cells were seeded into a 96-well plate at a density of 1 × 10^4^ cells/well and incubated for 24 h and then treated with BPS at various concentrations for 24 h. The cells were incubated for 30 min in the dark at 37 °C after adding 10 μL Cell Counting Kit-8 (CCK8) reagents (BS350A, Biosharp, Anhui, China) to each well. The OD value of each well was measured at a wavelength of 450 nm by a microplate reader (Thermo Scientific Varioskan LUX, Waltham, MA, USA). The result was expressed as the percentage of living cells, and the cell survival rate of the control group was regarded as 100%.

### 4.8. Immunofluorescence

The paraffin-embedded sections are first dewaxed and rehydrated, followed by antigen retrieval and blocking. Next, incubate with the primary antibody (GPX4, Abcam, AB125066, 1:200; CD86, CST, 19589, 1:400) at 4 °C overnight. After being washed with TBST, the fluorescent secondary antibody was used to cover the entire tissue, and then it was incubated at room temperature in the dark for 1 h. DAPI was added to stain the nucleus after washing with TBST. Finally, an anti-fluorescence quench agent was used to prevent fluorescence quenching, and then the sections were visualized under a fluorescence microscope (Nikon Eclipse C1, Nikon, Tokyo, Japan).

### 4.9. Determination of Oxidative Stress Index and Iron

Total proteins of hippocampal and cortical tissues were extracted using protein extraction kits, and the concentrations of hippocampus and cortex were detected using the Bicinchoninic acid (BCA) protein detection assay kit (P0010S, Beyotime, Shanghai, China). The levels of GSH (A006-2-1, Nanjing, China), MDA (A003-1, Nanjing, China), and Fe^2+^ (A039-2-1, Nanjing, China) in hippocampal and cortical tissues were detected strictly following the corresponding manufacturer’s instructions.

### 4.10. Reactive Oxygen Species Assay

Intracellular ROS levels in BV2 cells were detected using the dichlorodihydroflu-orescein diacetate (DCFH-DA) assay (CA1410, Solarbio, Beijing, China). Briefly, BV2 cells were seeded in 6-well plates and incubated for 24 h and then treated with BPS and Fer-1 for 24 h. Subsequently, 1 mL of DCFH-DA solution was added to each well and incubated at 37 °C for 20 min. Then, were washed cells three times with PBS (phosphate-buffered saline) and observed under a fluorescence microscope (Olympus IX73, Tokyo, Japan) with an excitation wavelength of 488 nm and an emission wavelength of 525 nm. ROS levels were quantified using ImageJ software 1.53a (NIH, Bethesda, MD, USA).

### 4.11. Real-Time Polymerase Chain Reaction (RT-qPCR)

The TRIzol method was used to extract total RNA from brain tissues, and the concentration and purity were assessed using a NanoDrop 2000 spectrometer (Thermo, Waltham, MA, USA). CDNA was synthesized using a reverse transcription kit (Monad, MR05101, Suzhou, China) and served as a template for RT-qPCR. The mRNA expression levels of *β-Actin*, *BDNF*, *PSD95*, *SYP*, *SYN1*, *GPX4*, *SCL7A11*, *TFR1*, *ACSL4*, *IL-17*, *IL-6*, *TNF-α*, *INF-γ*, and *IL-8* were quantified using a two-step fluorometer (Thermo, Waltham, MA, USA). The RT-qPCR conditions were as follows: pre-denaturation at 95 °C for 30 s, followed by 40 cycles of denaturation at 95 °C for 10 s, and annealing and elongation at 60 °C for 10 s. Relative gene expression levels were calculated using the 2^−∆∆Ct^ method. Primer sequences (Table 1) were synthesized by Wuhan Jin Kairui Company (Wuhan, China).

### 4.12. Western Blotting (WB)

The total protein of *mice* hippocampal and cortical tissues and BV2 cells were extracted by the PMSF method, and the concentrations were determined using a BCA kit. Proteins were separated via 12% or 10% sodium dodecyl sulfate-polyacrylamide gel electrophoresis (SDS-PAGE, G2042-4, Servicebio, Wuhan, China) and transferred to a polyvinylidene fluoride (PVDF, IPVH00010, Servicebio, Wuhan, China) membrane. The membrane was blocked at room temperature for 1 h and subsequently treated by primary antibodies such as β-Actin (Cat No. 81115-1-RR, 1:5000, Proteintech, Wuhan, China), BDNF (GB11559, 1:700, Servicebio, Wuhan, China), PSD95 (ab238135, 1:2000, Abcam), SYN1 (K110156P, 1:3000, Solarbio, Beijing, China), SYP (ab32127, 1:20,000, Abcam), TNF-α (GB113968, 1:1500 Servicebio, Wuhan, China), IL-17 (GB11110, 1:1000, Servicebio, Wuhan, China), IL-6 (GB11117, 1:1000, Servicebio, Wuhan, China), IBA1 (ab283319, 1:2000, Abcam), IL-8 (Cat No. 27095-1-AP, 1:2000, Proteintech, Wuhan, China), INF-γ (Cat No.15365-1-AP, 1:2000, Proteintech, Wuhan, China), GPX4 (ab125066, 1:5000, Abcam), SCL7A11 (DF12509, 1:2000, Affint, Rivoli Veronese, Italy), TFR1 (Cat No. 65236-1-Ig, 1:3000, Proteintech, Wuhan, China), and ACSL4 (ab155282, 1:30,000, Abcam) overnight at 4 °C. The following day, a goat anti-rabbit secondary antibody or a goat anti-mouse secondary antibody was added and incubated at room temperature on a shaker for 1 h. After washing with Tris-buffered saline with tween-20 (TBST, T8220, Solarbio, Beijing, China) three times, a chemiluminescence reagent and imaging system (ProteinSimplc, San Jose, CA, USA) was used to detect protein expression. Grayscale analysis was conducted with ImageJ software 1.53a.

### 4.13. Statistical Analysis

All experimental data were statistically analyzed and graphed using GraphPad Prism 8.0 software (San Diego, CA, USA). Data are presented as the mean ± standard deviation (SD) of at least three independent experiments. Statistical significance between groups was analyzed using Student’s *t*-test or one-way ANOVA. The difference was regarded as significant when *p* < 0.05.

## 5. Conclusions

In summary, this study demonstrated that exposure to BPS during pregnancy and lactation can lead to learning and memory impairments, as well as anxiety-like behaviors. Moreover, BPS may induce ferroptosis and oxidative stress in the brain and promote microglial activation and the release of pro-inflammatory mediators. Furthermore, the results indicated that Fer-1 effectively alleviates BPS-induced ferroptosis and inflammatory responses in microglia. Overall, these results provide valuable insights into potential strategies for intervening in BPS-induced neurodevelopmental impairments.

## Figures and Tables

**Table 1:** Primer sequences.

Genes		Sequences (5′–3′)
*BDNF*	Forward	GCCCATGAAAGAAGTAAACGTCC
	Reverse	AGTGTCAGCCAGTGATGTCGTC
*PSD95*	Forward	TCACATTGGAAAGGGGTAA
	Reverse	AAGATGGATGGGTCGTCA
*SYP*	Forward	CTTCGGCGACTTCTACTACTTT
	Reverse	GGAGCGGATGGATGTTTG
*SYN1*	Forward	CTTCTCGTCGCTGTCTAA
	Reverse	ATGGATCTTCTTCCCTTT
*GPX4*	Forward	ATTCTCAGCCAAGGACAT
	Reverse	ATGCCACAGGATTCCATACC
*SCL7A11*	Forward	TTGGAGCCCTGTCCTATGC
	Reverse	CGAGCAGTTCCACCCAGACC
*TFR1*	Forward	GAGTGGCTACCTGGGCTAT
	Reverse	TGTCTGTCTCCTCCGTTT
*ACSL4*	Forward	ACTTTCCACTTGTGACTTTAT
	Reverse	CTTCAGTTTGCTTTCCAG
*IL-17*	Forward	ACTACCTCAACCGTTCCA
	Reverse	GAGCTTCCCAGATCACAG
*IL-6*	Forward	TACCACTCCCAACAGACC
	Reverse	TTTCCACGATTTCCCAGA
*TNF-α*	Forward	GCGGTGCCTATGTCTCAG
	Reverse	TCACCCCGAAGTTCAGTA
*INF-γ*	Forward	AGCAACAACATAAGCGTC
	Reverse	CTCAAACTTGGCAATACTC
*IL-8*	Forward	GGACACTTTCTTGCTTGC
	Reverse	ATTACAGATTTATCCCCATT
*β-Actin*	Forward	GTGCTATGTTGCTCTAGACTTCG
	Reverse	ATGCCACAGGATTCCATACC

## Data Availability

The original contributions presented in this study are included in the article. Further inquiries can be directed to the corresponding author.
